# Solubilization of sulfuric acid lignin by ball mill treatment with excess amounts of organic compounds†

**DOI:** 10.1039/d2ra07235a

**Published:** 2023-01-04

**Authors:** Masatsugu Takada, Yutaka Okazaki, Haruo Kawamoto, Takashi Sagawa

**Affiliations:** a Graduate School of Energy Science, Kyoto University Yoshida-honmachi, Sakyo-ku Kyoto 806-8501 Japan takada-masatsugu@go.tuat.ac.jp; b Graduate School of Bio-Applications and Systems Engineering (BASE), Tokyo University of Agriculture and Technology 2-24-16, Nakacho Koganeishi Tokyo 184-8588 Japan okazaki.yutaka.8c@kyoto-u.ac.jp

## Abstract

In order to improve the solubility of sulfuric acid lignin (SL) in *N*,*N*-dimethylformamide (DMF), dry ball milling with excess amounts of additives such as l-tartaric acid was performed. Although the ball-milled SL without any additives was not soluble in DMF, when the SL was ball milled with an excessive amount of l-tartaric acid (the concentration of SL to be 0.1%), the dispersion and solubility of SL in DMF detected by the dynamic light scattering was greatly improved. Furthermore, the DMF solution showed clear photoluminescence, indicating that the distance between luminophores was modulated due to dispersion on the nanoscale. The structural analysis of the isolated lignin showed a decrease in molecular weight and the introduction of carboxylic acid groups. In other words, the introduction of hydrophilic functional groups into the lignin and simultaneously decrease in the molecular weight due to the cleavage of lignin linkages is considered to result in good dispersion in DMF on both the micro and macro scales. Similar effects were observed with the other chemicals containing several hydrophilic groups such as citric acid, d-glucose, and polyacrylic acid. Furthermore, this method is applicable to various lignins other than SL, and it is expected to utilize unused lignin resources.

## Abstract 2

Ball milling with excess amounts of additives enhanced the solubility of poor solubility lignins in DMF.

## Graphical abstract

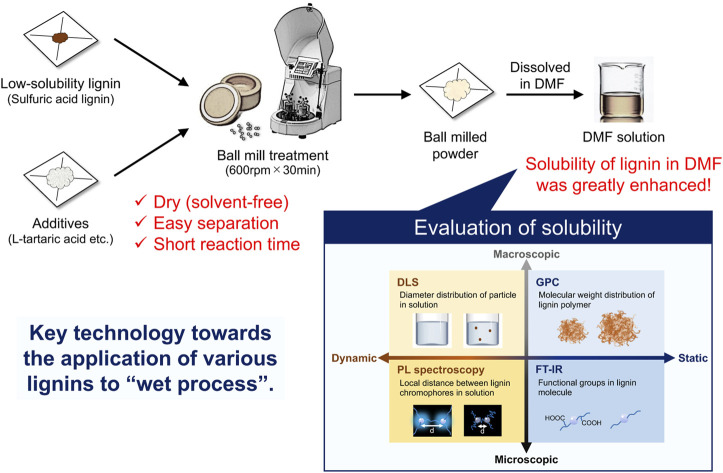

## Introduction

To achieve a sustainable society, carbon-neutral woody biomass resources have attracted attention as an alternative resource to unsustainable petroleum. Among the cell wall components of woody biomass, lignin, the most abundant aromatic polymer on earth, is expected to be a feedstock for valuable products. To create value-added materials, lignin needs to be soluble in solvents for commercial processes. In general, lignin is produced as a byproduct of biorefinery systems and chemical pulping processes. Regarding lignin from biorefinery systems, sulfuric acid lignin (SL), obtained by dilute or concentrated sulfuric acid hydrolysis to effectively produce monosaccharides,^1,2^ and enzymatic hydrolysis lignin, obtained by enzyme-mediated hydrolysis of biomass using microorganisms such as cellulase to convert polysaccharides to monosaccharides,^3^ are the major lignin byproducts. Both lignins show quite poor solubility in organic solvents owing to their high molecular weights and highly condensed structures. Furthermore, lignin is obtained as a byproduct in chemical pulping but condenses during the recovery and purification processes, resulting in decreased solubility in solvents.^4^ Such low-solubility lignins are mainly utilised as heat sources owing to their limited solubility. These low-solubility lignins could be used in various wet processes if they could be solubilized in organic solvents, resulting in their application in wide-ranging fields. To improve the solubility and dispersibility of low-solubility lignins, such as SL, which has very low solubility owing to their highly condensed structure, various structural modifications by chemical treatment have been attempted. For example, lignin solubilization has been reported using an alkaline aqueous solution or hydrous dioxane solution using phenolization under acid catalysis,^5^ sulfomethylation or arylsulfonation,^6,7^ carboxylation using monochloroacetic acid or amino acids,^8^ and by introducing carboxy groups by alkaline hydrothermal treatment.^9^ All these methods require higher amounts of reagents and long reaction times and have waste problems, such as waste fluid treatment owing to wet processing. Therefore, dry processes to modify the lignin structure quickly and cheaply are greatly needed.

In recent years, mechanochemical treatment, represented by ball milling, has attracted attention in organic chemical reactions. For example, carbon–carbon bond formation,^10^ heterocycle synthesis,^11,12^ protecting group synthesis,^13^ reduction reaction control,^14^ and natural polymer functionalization^15^ have been reported. Regarding polymeric compounds, the formation of new bonds by the generated mechanoradicals is known to occur simultaneously with the decrease in molecular weight caused by the cleavage of polymer chains and is used in the synthesis of copolymers.^16^

**Scheme 1:**
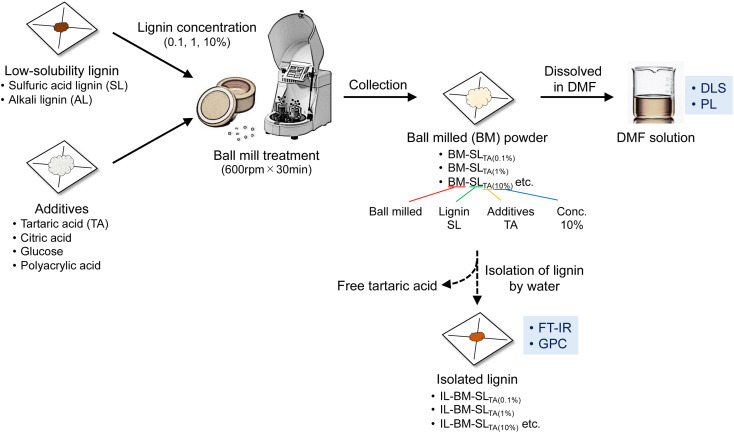
Overview of experimental procedures used in this study.

**Scheme 2:**
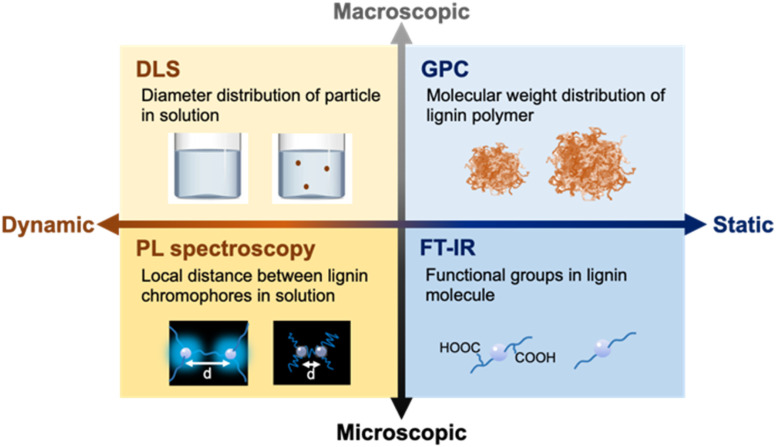
Evaluation methods used in this study from both dynamic and static perspectives at microscopic and macroscopic scales.

In this study, this mechanoradical reaction generated by ball mill treatment was used to react low-solubility lignin (SL) with excess amounts of additives, with the aim to cleave crosslinking bonds in lignin molecules and introduce solvophilic functional groups using organic capping reagents, as shown in Scheme 1. Tartaric acid was first selected as an additive because it contains hydrophilic functional groups, such as carboxylic acids and hydroxyl groups. To evaluate the lignin solubility and understand the solubilization behavior, various techniques, from both dynamic and static viewpoints at microscopic and macroscopic levels, were used, including dynamic light scattering (DLS), photoluminescence (PL) spectroscopy, gel permeation chromatography (GPC), and Fourier-transform infrared (FT-IR) spectroscopy (Scheme 2).

## Experimental

### Materials and chemicals

The sapwood of Japanese cedar (*Cryptomeria japonica*) was milled using a Wiley mill (Thomas Scientific, NJ), and the obtained flour was sieved with mesh screens to collect particles with sizes of 0.15–1.0 mm. The sieved flour was extracted with acetone in a Soxhlet apparatus and dried at 105 °C for 24 h prior to the experiments. Sulfuric acid lignin (SL) was prepared from the extractive-free flour by following the typical Klason method.^17^ Alkali lignin (AL) and all chemicals used in this study were of reagent grade, purchased from Nacalai Tesque, Inc. (Kyoto, Japan), and used without further purification.

### Ball milling treatment

SL or AL was mixed with an excess amount of additive in different lignin-additive concentration ratios (0.1%, 1%, and 10%). The mixtures were placed in a zirconia container and milled using a planetary ball mill (Pulverisette 7S, Fritsch Japan Co., Ltd, Japan) for 10 min at 600 rpm, performed three times with 15 min cooling intervals. For comparison, lignin without additives was also ball milled. Regarding the notation used for ball-milled (BM) powders, when SL was mixed with l-tartaric acid (TA) at a lignin concentration of 10%, the resulting BM powder was denoted as BM-SL_TA(10%)_, as shown in Scheme 1.

### Analysis of BM powder DMF solution

The BM powder was dissolved in DMF to a lignin concentration of 0.1 mg mL^−1^. The solubility of the BM powder in DMF was evaluated by DLS using a *ζ*-potential and particle-size analyzer (ELSZ2000ZS; Otsuka Electronics Co., Ltd, Osaka, Japan). Furthermore, UV-Vis absorption and PL spectra were recorded using V-650 (Jasco Co., Ltd, Tokyo, Japan) and FP-8600 (Jasco Co., Ltd, Tokyo, Japan) instruments, respectively.

### Characterization of isolated lignin

To characterize the lignin structure of BM powder from SL and l-tartaric acid, lignin was isolated using a poor solvent, water. Regarding the notation used for the isolated lignin, the lignin isolated from BM-SL_TA(10%)_ was denoted as IL-BM-SL_TA(10%)_, as shown in Scheme 1. The isolated lignin was analyzed using FT-IR spectroscopy and GPC. FT-IR spectra were recorded using the KBr pellet method (IRAffinity-1S instrument, Shimadzu Co., Kyoto, Japan). GPC was performed using an LC-10A instrument (Shimadzu, Kyoto, Japan) under the following conditions: Column, Shodex KF-801 + KF-802 + KF-802.5 + KF-803 (Showa Denko, Tokyo, Japan); eluent, tetrahydrofuran (THF); flow rate, 0.6 mL min^−1^; column temperature, 50 °C; detector, UV light at 280 nm. For comparison, polystyrene standards (molecular weights (MWs) of 162, 580, 1270, 2960, and 5000) were used. Prior to GPC analysis, lignin was acetylated to improve its solubility in THF in accordance with the general method.^18^

## Results and discussion

### Ball milling of sulfuric acid lignin with tartaric acid

**Fig. 1:**
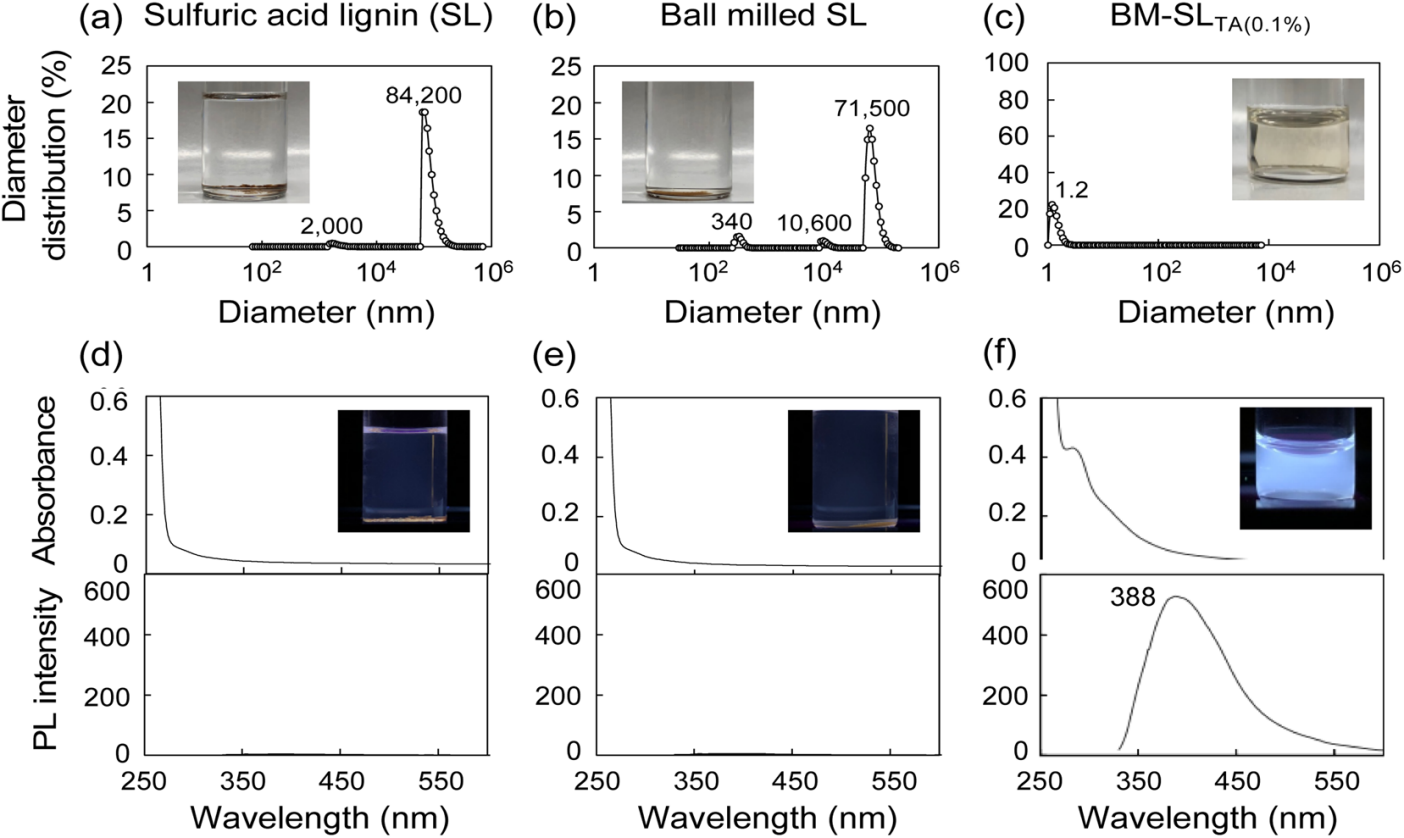
(a–c) Diameter distribution of (a) sulfuric acid lignin (SL), (b) ball milled SL, and (c) BM-SL_TA(0.1%)_ in DMF solution detected by DLS. The concentration of the solution is standardized to be 0.1 mg mL^−1^ of lignin. (d–f) Their UV-Vis absorbance and PL spectra excited at 320 nm. The inserted pictures were taken under the irradiation of UV light at 365 nm.

When SL and ball-milled SL were added to DMF, both powders did not dissolve in DMF and no color was observed (Fig. 1(a) and (b)). In contrast, when BM-SL_TA(0.1%)_ was dissolved in DMF, a yellowish-colored transparent solution was obtained (Fig. 1(c)), which was derived from the dissolved lignin. Furthermore, DLS analysis was conducted on the DMF solutions to detect the particle diameter distribution (Fig. 1(a)–(c)). Although ball milling treatment changed the diameter distribution, even ball-milled SL showed large-diameter particles. However, for BM-SL_TA(0.1%)_, no large particles were detected in the DMF solution, indicating that the dispersion and solubilization of SL in DMF were greatly enhanced (Fig. 1(c)). Furthermore, when the UV-Vis and PL spectra of the solutions were analyzed, only the solution of BM-SL_TA(0.1%)_ clearly showed the UV absorbance and PL peak at 388 nm. Regarding the PL properties of lignin in the solution, lignin PL was observed only when the distance between chromophores was sufficiently maintained, and PL quenching occurred when the distance between the chromophores was too small.^19^ Therefore, BM-SL_TA(0.1%)_ was well dispersed at the molecular level. When the ball-milled SL was added to DMF with l-tartaric acid of 100 mg mL^−1^, the ball-milled SL powder did not dissolve in DMF, as shown in Fig. S1.† These results indicate that the synergistic effect between the existence of the excess amounts of additives (*e.g.*l-tartaric acid) and ball-milling is dominant to solubilizing SL at the molecular level rather than the acidity of l-tartaric acid.

### Influence of lignin on tartaric acid concentration ratio on solubility

**Fig. 2:**
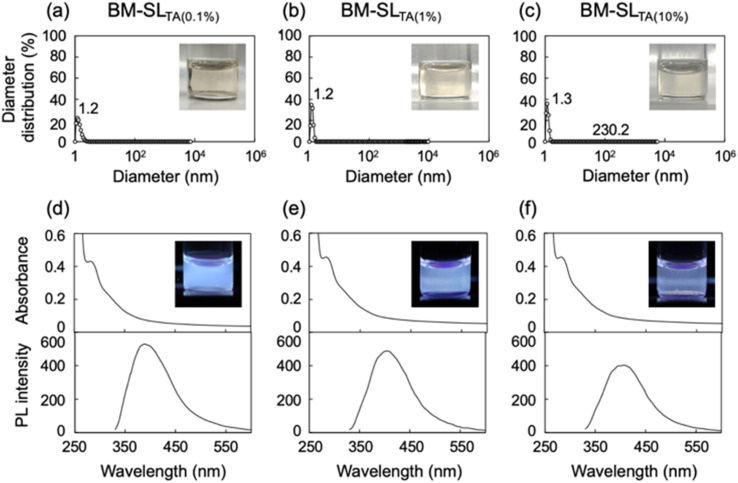
(a–c) Diameter distribution of SL ball milled with l-tartaric acid at different lignin to l-tartaric acid ratios of (a) 0.1%, (b) 1%, and (c) 10%. Samples were dissolved in DMF at lignin concentrations of 0.1 mg mL^−1^. (d–f) Corresponding UV-Vis absorbance and PL spectra excited at 320 nm. Inserted images were recorded under the irradiation of UV light at 365 nm.

The influence of the lignin to l-tartaric acid concentration ratio on the solubility was examined, as shown in Fig. 2. With an increasing ratio of SL to l-tartaric acid, the 1% mixture dissolved well in DMF, in a manner similar to the 0.1% mixture (Fig. 2(b)). Although the 10% mixture was partially dissolved in DMF, large particles were also detected. From PL spectra analysis, the 1% mixture showed a PL intensity as high as that of the 0.1% mixture, but PL quenching was observed in the 10% mixture (Fig. 2(f)). Therefore, based on the results of DLS and PL analysis, the enhancing effect of ball milling with l-tartaric acid on lignin solubility was limited in the 10% mixture. Therefore, an excess amount of the reaction medium, namely, l-tartaric acid, was required for the desired reaction.

### Characterization of isolated lignin

**Fig. 3:**
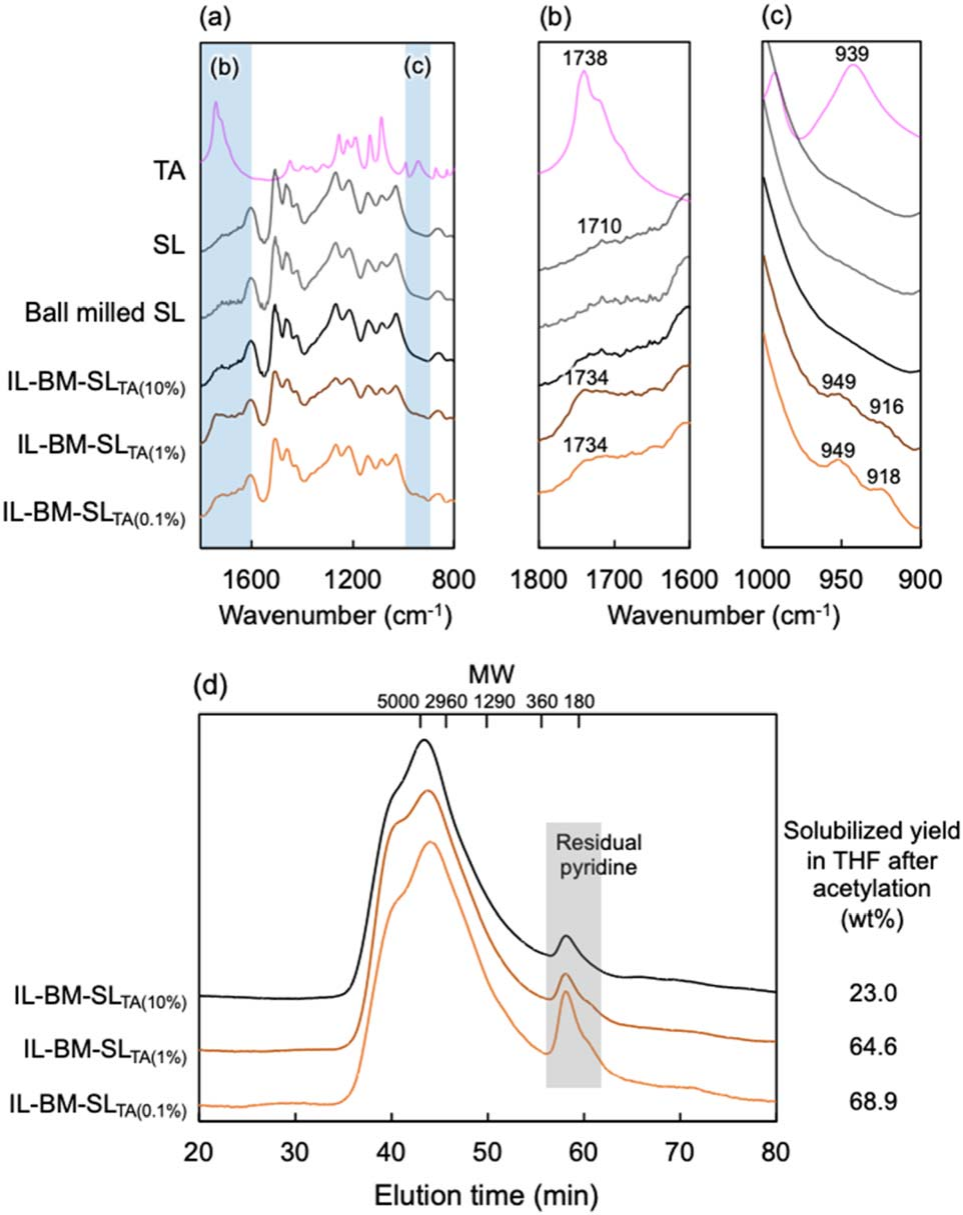
(a–c) FT-IR spectra of isolated lignins (a)1800–800 cm^−1^, (b) 1800–1600 cm^−1^, (c) 1000–900 cm^−1^. (d) The molecular weight distribution of their acetylated samples dissolved detected by GPC.

To characterize the lignin structure of BM-SL_TA_, lignin was isolated using water, a poor solvent, to remove tartaric acid. The isolated lignin (IL-BM-SL_TA_) was characterized by FT-IR and GPC, as shown in Fig. 3. As a result, IL-BM-SL_TA(0.1%)_, IL-BM-SL_TA(1%)_, and IL-BM-SL_TA(10%)_ showed IR spectra similar to SL and ball-milled SL, indicating that mechanical ball milling treatment did not drastically alter the lignin structure. However, a new peak at 1730 cm^−1^, which originated from the carbonyl group,^20^ appeared only in lignins isolated from the 0.1% and 1% mixtures (Fig. 3(b)). New peaks also appeared at 920 and 950 cm^−1^, which originated from O–H bending (Fig. 3(c)). Although these peaks were considered to originate from carboxylic acid groups, the peak positions were different from those in l-tartaric acid, indicating that these peaks were not derived from the free tartaric acid. Therefore, the carboxylic acid structure was clearly incorporated in the lignin molecules of IL-BM-SL_TA(0.1%)_ and IL-BM-SL_TA(1%)_.

The molecular weight distribution was evaluated by GPC analysis (Fig. 3(d)). Prior to GPC analysis, the isolated lignins were acetylated to improve their solubility in THF. As a result, SL and ball-milled SL did not dissolve in THF. IL-BM-SL_TA(0.1%)_ and IL-BM-SL_TA(1%)_ were two-thirds dissolved in THF, with the soluble portion mainly composed of molecular weights ranging from 5000 to several tens of thousands. In contrast, the solubility of IL-BM-SL_TA(10%)_ in THF was limited (23%), indicating that its molecular weight was higher than those from 0.1% and 1% mixtures.

Based on this evidence, ball milling with an excess amount of l-tartaric acid led to the reduction of the lignin molecular weight and the introduction of carboxylic acid groups. The introduction of carboxylic acid groups by the generated mechanoradicals occurred simultaneously with a decrease in molecular weight owing to the cleavage of the lignin intermolecular linkages. Consequently, lignin was well dispersed in DMF on both molecular and macroscopic scales.

### Comparison of solubility using other organic compounds

**Fig. 4:**
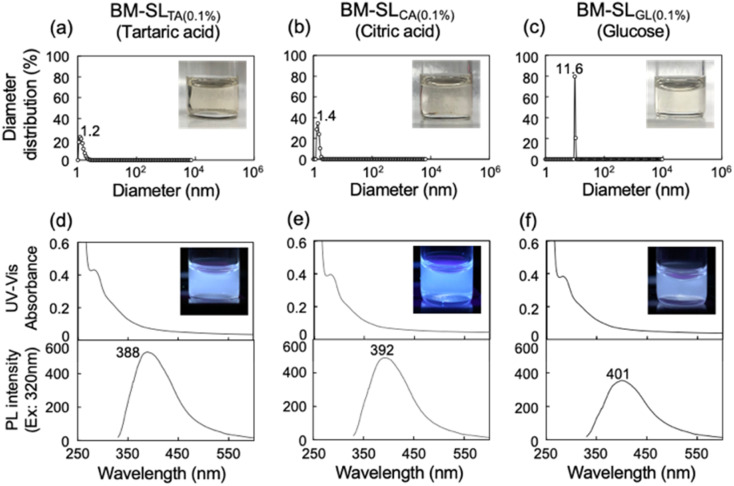
(a–c) Diameter distribution of SL ball milled with (a) tartaric acid, (b) citric acid, and (c) glucose, dissolved in DMF at a lignin concentration of 0.1 mg mL^−1^. (d–f) Corresponding UV-Vis absorbance and PL spectra excited at 320 nm. Inserted images were recorded under the irradiation of UV light at 365 nm.

Several organic chemicals other than l-tartaric acid were used as media, including citric acid and d-glucose, as shown in Fig. 4. DLS analysis of DMF solutions of all samples showed that no large-diameter particles were present. Furthermore, all solutions clearly showed PL, indicating that the distance between the chromophores was sufficiently maintained. Therefore, SL was dissolved and dispersed in DMF on both molecular and macroscopic scales. Similar solubilization was achieved when a polymer, polyacrylic acid, was used as an additive (Fig. S2†). As both low molecular weight compounds and polymeric compounds can be used as additives, various additives can be expected to be applicable to this solubilization technique using the ball milling treatment.

### Application to commercial alkali lignin

**Fig. 5:**
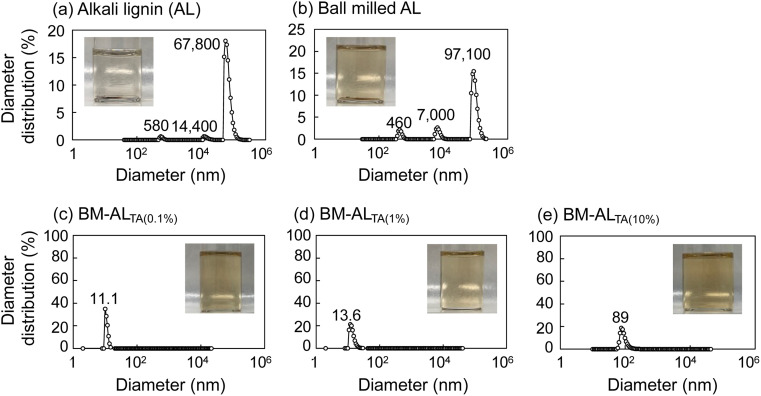
Diameter distribution of (a) alkali lignin (AL), (b) ball-milled AL, and (c–e) AL ball milled with different ratios of l-tartaric acid. Lignin concentrations are (c) 0.1%, (d) 1%, and (e) 10%. Samples are dissolved in DMF at a lignin concentration of 0.1 mg mL^−1^.

Next, this ball milling solubilization technique was applied to commercial AL. Images of AL and ball-milled AL in DMF, and their diameter distributions detected by DLS analysis, are shown in Fig. 5. Both the lignins did not dissolve in DMF. However, when AL was milled with l-tartaric acid at lignin ratios of 0.1%, 1%, and 10%, no large particles were detected in the DMF solution of the resulting product, indicating that the dispersion and solubilization of AL in DMF was greatly enhanced (Fig. 5(c)–(e)). Therefore, this technique can be applied to various lignins. Regarding the solvents, the dissolution of SL and AL in dimethyl sulfoxide (DMSO) was also confirmed, but, other than DMF, some typical solvents such as ethanol and methanol showed limited effects.

## Conclusions

In this study, the solubility of lignins, such as SL and AL, in DMF was successfully improved by ball milling with excess amounts of organic compounds, such as l-tartaric acid. During ball milling treatment, the introduction of carboxylic acid groups by the generated mechanoradicals occurred simultaneously with a decrease in molecular weight owing to the cleavage of the lignin intermolecular linkages. Consequently, the lignins were well dispersed in DMF at both micro- and macroscopic scales. Furthermore, lignin solubilization was achieved using other organic compounds, such as citric acid, d-glucose, and polyacrylic acid. Although in this work, we focused on the usage of several compounds and solvents, the proposed concept has the potential to accept a wide variety of compounds and solvents, including greener chemicals such as ethanol, ethyl acetate, and acetonitrile, theoretically. Based on these lines of evidence, this system is expected to become a key technology for the effective utilization of unused lignin resources.

## Conflicts of interest

There are no conflicts to declare.

## Supplementary Material

RA-013-D2RA07235A-s001
