# Supplementary material for: Solubilization of sulfuric acid lignin by ball mill treatment with excess amounts of organic compounds
Source: RSC Adv. 2023 Jan 4;13(2):1059–65. doi: 10.1039/d2ra07235a (PMC9811933; doi:10.1039/d2ra07235a)
Supplement: RA-013-D2RA07235A-s001 [file RA-013-D2RA07235A-s001.pdf]

Electronic Supplementary Material (ESI) for RSC Advances.
This journal is © The Royal Society of Chemistry 2022

Supporting Information for

Solubilization of sulfuric acid lignin by ball mill treatment with excess amounts of organic compounds

Masatsugu Takada,*[a,b] Yutaka Okazaki,*[a], Haruo Kawamoto,[a] and Takashi Sagawa[a]

a Graduate School of Energy Science, Kyoto University, Yoshida-Honmachi, Kyoto 606-8501, Japan

b Graduate School of Bio-Applications and Systems Engineering (BASE), Tokyo University of Agriculture and Technology, 2-24-16, Nakacho Koganeishi, Tokyo 184-8588, Japan

*Corresponding authors:

Dr. M. Takada; takada-masatsugu@go.tuat.ac.jp

Dr. Y. Okazaki; okazaki.yutaka.8c@kyoto-u.ac.jp

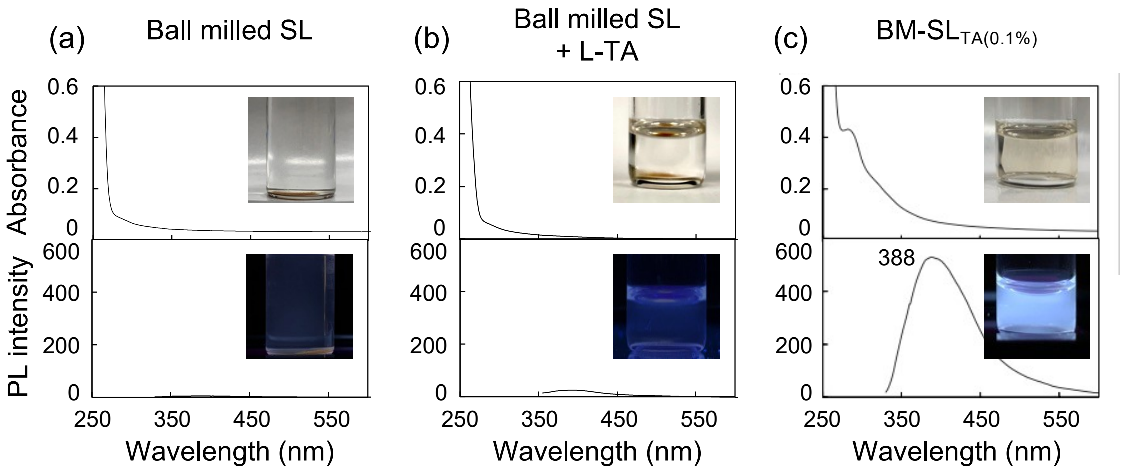

Figure S1 Pictures of (a) ball milled sulfuric acid lignin (SL) DMF solution at 0.1 mg mL^{-1}, (b) DMF solution of ball milled SL at 0.1 mg mL^{-1} with addition of L-tartaric acid (L-TA) at 100 mg mL^{-1}, and (c) DMF solution of BM-SL$_{TA(0.1\%)}$ at 0.1 mg mL^{-1}, and their UV-Vis absorbance and PL spectra excited at 320 nm. The inserted pictures as indicated below were taken under the irradiation of UV light at 365 nm.

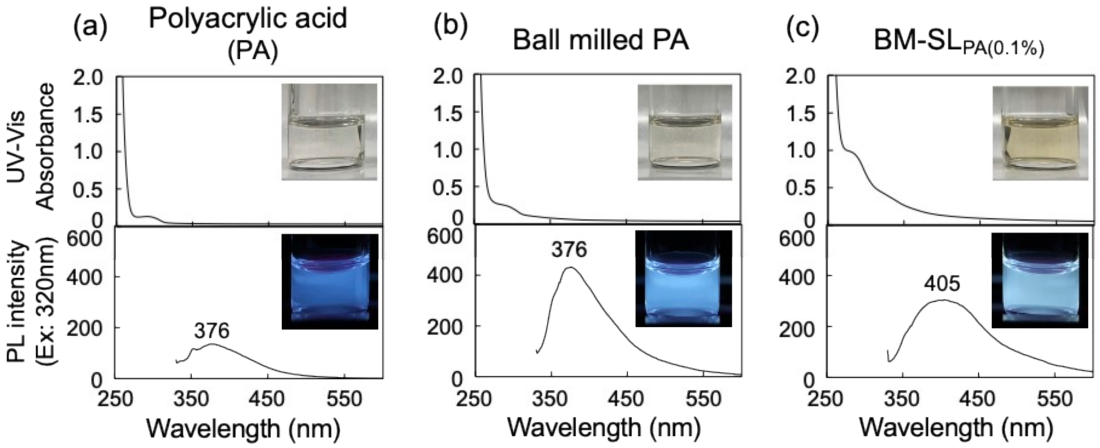

Figure S2 Pictures of (a) polyacrylic acid (PA), (b) ball milled PA, and (c) BM-SL$_{PA(0.1\%)}$, and their UV-Vis absorbance and PL spectra excited at 320 nm. The inserted pictures as indicated below were taken under the irradiation of UV light at 365 nm.
